# Bordered Pit Formation in Cell Walls of Spruce Tracheids

**DOI:** 10.3390/plants10091968

**Published:** 2021-09-21

**Authors:** Dmitry G. Chukhchin, Ksenia Vashukova, Evgeniy Novozhilov

**Affiliations:** Department of Biology, Ecology and Biotechnology, Northern (Arctic) Federal University, Northern Dvina Embankment 17, 163002 Arkhangelsk, Russia; dimatsch@mail.ru (D.G.C.); e.novozhilov@narfu.ru (E.N.)

**Keywords:** pit formation, tracheids, borded pit, exosomes, endoglucanases, cell wall structure

## Abstract

The process of pit formation in plants still has various questions unaddressed and unknown, which opens up many interesting and new research opportunities. The aim of this work was elucidation of the mechanism for the formation of bordered pits of the spruce (*Picea abies* (L.) Karst.) tracheid with exosomes participation and mechanical deformation of the cell wall. Sample sections were prepared from spruce stem samples after cryomechanical destruction with liquid nitrogen. The study methods included scanning electron microscopy and enzymatic treatment. Enzymatic treatment of the elements of the bordered pit made it possible to clarify the localization of cellulose and pectin. SEM images of intermediate stages of bordered pit formation in the radial and tangential directions were obtained. An asynchronous mechanism of formation of bordered-pit pairs in tracheids is proposed. The formation of the pit pair begins from the side of the initiator cell and is associated with enzymatic hydrolysis of the secondary cell wall and subsequent mechanical deformation of the primary cell walls. Enzymatic hydrolysis of the S_1_ layer of the secondary cell wall is carried out by exosome-delivered endoglucanases.

## 1. Introduction

Gymnosperm wood is superficially simple, composed mostly of single-celled tracheid. Tracheids perform conductive and mechanical functions. The intertracheid pits are quite specialized and have the most complex structure. These are usually circular bordered pits with a torus-margo pit membrane [1]. In the process of biogenesis, tracheids go through all the stages characteristic of most plant cells: initiation, cell growth by stretching, differentiation, ageing, and death.

The traditional concept of the structure of the tracheid cell wall includes the allocation of two main structural parts—the P primary wall, adjacent to the middle lamella, and the S secondary wall. During the thickening of the cell wall, a secondary wall is formed, consisting of three layers: outer S_1_, middle S_2,_ and inner S_3_. These layers differ in the orientation of microfibrils, which is estimated by the angle of inclination in relation to the longitudinal axis of the cell; the microfibrils lie almost parallel to each other inside each layer. In the S_1_ layer, cellulose microfibrils are located almost perpendicular to the longitudinal axis of the cell, which limits its radial extension. The thickness of this layer is comparable to the thickness of the primary cell wall—approximately 0.10–0.35 µm (5–10% of the total cell wall thickness). The S_1_ layer of the spruce tracheids is rather uniform and hard [2]. The S_2_ layer forms the main part of the cell wall. Microfibrils follow steep spirals at an angle to the axis of the tracheid. The secondary cell wall has a similar structure in cells of different types such as fibres and tracheids in numerous plant species that are evolutionarily and taxonomically distant. The prevalence of its structure and stability during evolution are most likely indicative of its main task—to withstand enormous mechanical stresses while maintaining flexibility [3].

Tracheids and associated pits perform important functions for the plant: they provide water flow between the conducting elements and prevent air from entering functional and water-conducting elements. Each pit has a pit chamber. Both chambers are separated from each other by a thin section of the cell wall—a pit closing film, or pit membrane. The pit membrane consists of two primary cell walls and a middle lamella between them. In the tracheids of gymnosperms, the pit membrane that separates the bordered-pit pair often has a thickening called the torus. The flexible margo consists of cellulose microfibrils located in a circle that extend from the edge of the torus to the inner circumference of the pit border. Since the pit membrane is elastic, at a certain pressure, the torus is displaced to one or the other side of the border, closing the pit opening (aperture) since its dimensions are larger. In this position, the pit does not participate in the conductance of substances and is considered closed. Thus, the bordered pits function as valves to prevent air from entering the conductive tracheids, which could disrupt the continuity of the water column [4]. The torus-margo structure is important for maximizing the conductivity of the inherently length-limited tracheid [1].

There are hypotheses about the formation of pits in the cell walls of plants.

Within the concept that desmotubules traversing the compound middle lamella enable intercellular communication, such as that presumed necessary for the formation of symmetrical pit pairs, the hypothesis that bordered pits develop where «pit fields» of plasmodesmata exist has been advocated [5]. Plasmodesmata are located mainly in the primary pit fields (a thin, but not ruptured, section of the primary cell wall) and then remain in the pit membrane [6]. According to this hypothesis, to form a pit in the cell wall, enzymatic hydrolysis of the noncellulose matrix, consisting of pectins, hemicelluloses, and a small number of proteins, is sufficient. What remains is the pit membrane, which is a network of cellulose microfibrils of the primary walls of two cells.

The main tenets of this hypothesis are widely used when discussing research results; however, the existing theoretical concepts of the pit formation system cannot provide answers to a number of fundamental questions. How are such primary pit fields formed in very thin primary cell walls? Why does the location of the primary pit fields coincide in two adjacent anatomical elements, including elements of different structures (rays, tracheids, axial parenchyma), which, moreover, at different stages of their development go through stages of compression, expansion, and elongation?

Savidge (2014) [7] raised the following questions regarding the features of the formation of bordered pits: «Why should the general S_1_ layer merely abut the bordered-pit rim, rather than override it as the S_2_ and S_3_ layers do? How do the S_2_ and S_3_ layers form an overarching border? Why when forming the overarching border, do the S_2_ and S_3_ layers not entirely seal off the structure, such that a perfectly circular opening—the aperture—is produced?» All these questions indicate that the well-known hypothesis is poorly substantiated. Savidge (2014) [7] proposed a mechanism for the formation of a bordered pit torus with the participation of special organelles. It is not known where the special organelles originate. The process of pit formation in neighbouring cells is synchronous [7]. How the appearance of special organelles opposite each other is synchronized has not been explained.

Barnett and Harris (1975) [8] and Barnett (1982) [9] also considered the formation of primary pit fields, as sites of future pit localization, necessary; however they showed that plasmodesmata are absent during pit formation in tracheids. In their opinion, the development of the primary pit field in the tracheids occurred under the action of a certain agent without explanation whether the agent was in the primary wall or was delivered by vesicles. The fusion of multivesicular bodies (MVBs) with a plasma membrane results in the release of small vesicles into the extracellular spaces of fungi and higher plants [10,11,12]. Extracellular vesicles (EVs) visualized on the internal layers of the cell walls of woody plants proved to be exosomes based on their diameter (65–145 nm) [13].

Currently, another hypothesis has become more widespread—the pit formation with the participation of Rho-like GTPases from plant (ROP) and cortical microtubules [14,15,16,17,18,19,20,21,22]. Microtubules have been characterized as essential components of the cell division apparatus. In plant cells, nucleation of new microtubules occurs at dispersed sites at the cell cortex, the area that is in very close proximity to the plasma membrane within the cell. The behavior of cortical microtubules determines the overall organization of the cortical microtubule array and thereby determines the asymmetric cell growth of plant cells [23].

GTPases regulate the behavior of the cytoskeleton through various cellular events. ROP in plants control the cell wall deposition pattern by governing the behavior of microtubules and actin filaments, which thereby determines cell shapes and functions. However, how specialized domains are established in cell walls with edges and boundaries through the action of ROP signaling remains poorly understood [20]. The dynamic rearrangement of the microtubules and actin filaments have also been recognized in the cultured Arabidopsis thaliana cells differentiating into tracheary elements in vitro [24]. It is believed [18,19,21,25] that during the development of Arabidopsis xylem, cell wall deposition is locally inhibited to form pits. Rho-like GTPase from plant 11 (ROP11) is locally activated to induce microtubule disassembly through its effector, MICROTUBULE DEPLETION DOMAIN 1(MIDD1), and Kinesin-13A, resulting in the formation of pits. During pit formation, bordered cell walls specifically develop at the boundary of pits. ROP11, MIDD1, Kinesin-13A, and IQ67 DOMAIN PROTEIN 13 (IQD13) combined action excludes microtubule-guided cellulose synthases during secondary cell wall formation specifically from a plasma membrane region which then gives rise to a pit. However, little is known about how the distinct boundaries of pits are established along with ROP11-MIDD1-dependent pit formation [20]. Despite the observation that cellulose microfibrils co-align with cortical microtubules, mechanistic details regarding how microtubules and cellulose microfibrils work together to effect cell expansion are lacking [23].

In the gymnosperms tracheids the process of pit formation proceeds in a unique way since the creation of a pit occurs in anatomical elements that have not only a primary wall but also several layers of a secondary wall. The intertracheid pits of gymnosperms are quite specialized. Unlike gymnosperms, the pit membrane of angiosperms is generally homogenous in thickness and porosity, and it is a much simpler valve [1]. When studying pits, traditional methods such as optical microscopy, TEM and SEM are often complemented by techniques such as cryo-SEM [26] and AFM [27]. Enzymes are used to open the pit structure [28]. Based on our analysis of published data as well as our preliminary experiments, pits in rays, vessels, tracheids, and fibres are formed in different ways by different mechanisms. This article will focus on pit formation between two tracheids. The aim of this work was elucidation of the mechanism for the formation of bordered pits of the spruce tracheid with exosomes participation and mechanical deformation of the cell wall.

## 2. Results

### 2.1. Enzymatic Treatment of Bordered Pits in Spruce Tracheids

**Figure 1:**
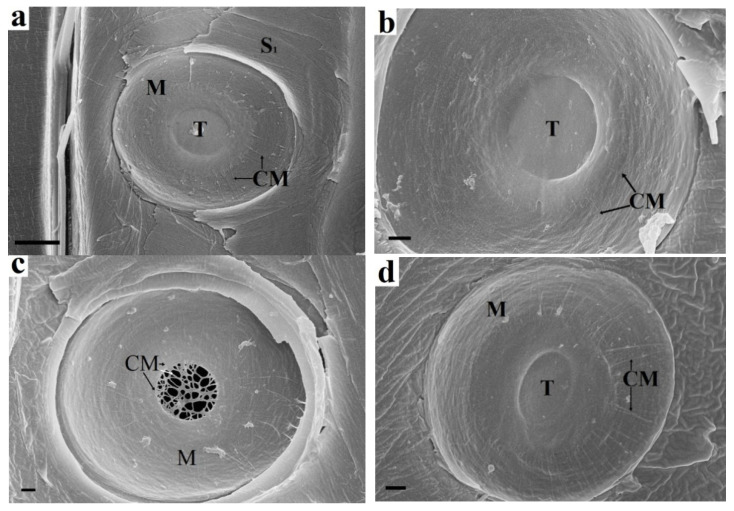
Enzymatic treatment of spruce bordered pit elements: (**a**) Untreated sample. The torus fits snugly against the border. Margo microfibrils emerge from the torus and reach the pit periphery. (**b**) Endoglucanase treatment. The margo microfibrils are absent at the pit periphery but are preserved near the torus. The torus is also intact and is held by adhesion to the border. Arrows indicate the ends of microfibrils. (**c**) Pectinase treatment. The torus is hydrolysed, and only the matrix microfibrils are visible, which are a natural continuation of the margo microfibrils. (**d**) Xylanase treatment. The torus, margo, and border are visibly unchanged. CM cellulose microfbrils, S_1_ S_1_ layer, M margo, T torus. Bars 5 µm (**a**), 1 µm (**b**–**d**).

Determination of the mechanism of pit formation is impossible without knowledge of the chemical composition of their parts. It follows from Figure 1b that cellulose microfibrils of margo near the torus are protected by cell wall constituents from contact with endoglucanase and remain unchanged. The same microfibrils at the pit periphery lack such protection and are hydrolysed. Not a single microfibril emanating from the torus reached the periphery of the pit. Thus, microfibrils near the torus are most likely covered with pectin or xyloglucan.

Pectinase treatment (Figure 1c) results in hydrolysis of torus pectin. The microfibrils that make up the torus framework are visible. They are a natural extension of margo microfibrils. The random arrangement of microfibrils and the presence of pectin indicate that the torus is part of the preserved P layers of adjacent cells. Margo microfibrils are also P layer microfibrils; however, they have been deformed. Their orientation from the torus to the pit periphery is due to mechanical action on the P layer during pit formation. Most likely, the P layers are bent in the form of a hemisphere during the formation of a bordered pit. In this case, the bending and stretching of the P layer at the pit periphery is maximal, while that of the torus is minimal. This also explains the rounded shape of the pits. The microfibrils are pulled out of the pectin matrix when the P layer is deformed. This leads to the loss of the protective effect of pectin, as shown in Figure 1b. Xylan is not a structure-forming material of the pit elements. The torus, margo, and border are visibly unchanged (Figure 1d).

### 2.2. Visualization of the Bordered Pits Formation in Spruce Tracheids 

**Figure 2:**
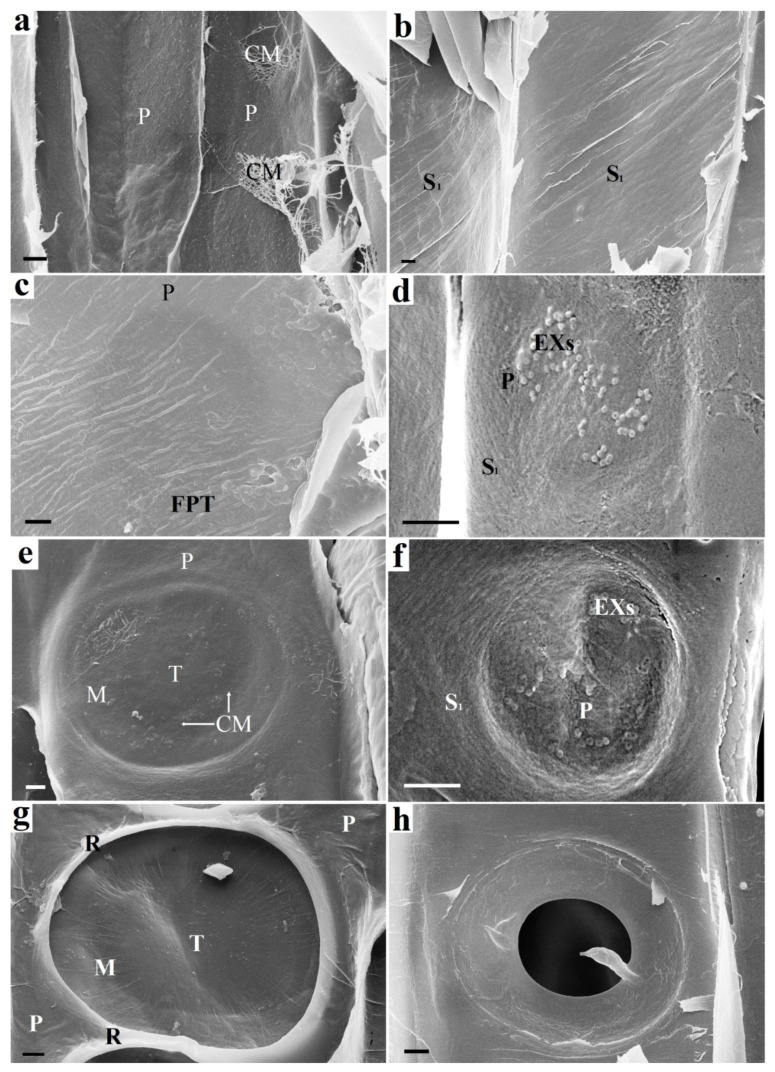
The beginning of the bordered pit formation: on the left (**a**,**c**,**e**,**g**)—following pit tracheid (FPT) and on the right (**b**,**d**,**f**,**h**)—starting pit tracheid (SPT): (**a**) Cell walls of adjacent tracheids (SPT and FPT) before pit formation. Only the P layer is present. (**b**) SPT cell wall prior to pit formation. The inner surface is already covered with an S_1_ layer. (**c**) FPT cell wall. Round darkening is observed under the surface of the FPT P layer, which indicates the destruction of the material on the opposite side (destruction of the S_1_ layer of the SPT). (**d**) Exosomes visible on the surface of the S_1_ layer of the SPT. (**e**) FPT P layer after deformation caused by pressure difference in adjacent SPT and FPT tracheids. Areas with stretched microfibrils are visible in the area of the margo and torus. (**f**) Destruction of the SPT S_1_ layer at the site of exosome localization. The S_1_ layer is partially destroyed. The P layer is visible under it. (**g**) An S_1_ layer begins to form on the FPT surface. The formed torus and margo of the bordered pit are observed. The rim is bordered by the rims of adjacent pits. (**h**) The pit border partially formed by the S_2_ layer on the SPT surface. Further border formation during the S_2_ and S_3_ layer deposition makes the SPT and FPT pits appear the same. *CM*—cellulose microfbrils, *EX—* exosomes, *S*_1_—S_1_ layer, *P*—P layer, *M*—margo, *T*—torus, *R*—rim, *FPT*—following pit tracheid. Bars 1 µm (**a**,**d**,**e**–**g**), 2 µm (**b**,**c**,**h**).

Figure 2 shows a visualization of the formation stages of a tracheid bordered pit. There is only an elastic P layer (Figure 2a) before the tracheid reaches its final size (maximum length and width). The expansion of tracheid is carried out by osmotic pressure. The cell wall becomes more durable when the outer S_1_ layer of the secondary cell wall is formed. This layer is deposited from the lumen side of the tracheid on the P primary wall using only the carbohydrates stored by the cell, mainly starch. The pits, primary pit fields, and cavities are absent in the S_1_ layer. The cellulose microfibrils of the S_1_ layer are densely packed and located at an angle to the tracheid axis (Figure 2b). This is a characteristic feature of the S_1_ layer by which it can be easily distinguished from other layers of the cell wall. The spaces between microfibrils are filled with hemicelluloses and lignin precursors [29]. 

The entry of the substrate into the tracheid from the outside is unlikely, since there are still no pits in it. Enzymatic hydrolysis of the S_1_ layer proceeds with the participation of exosome-delivered endoglucanases (Figure 2d). The S_1_ layer is deformed and destroyed at the exosome localization. The destruction of the S_1_ layer makes the P layer visible (Figure 2f). Bordered pit pairs of adjacent tracheids develop asynchronously. Thinning of the material occurs due to the S_1_ layer hydrolysis, and the site of pit localization in the adjacent tracheid looks like a darker circular area (Figure 2c). This indicates the destruction of the material from the other side. Thinning of the S_1_ layer leads to deformation of the (P layer + middle lamella + P layer) complex caused by a pressure drop in adjacent tracheids. The result of the deformation is shown in Figure 2e. The torus and margo of the intertracheid bordered pit are formed. The microfibrils of the S_1_ layer form rims (Figure 2g). Layer S_1_ begins to form on the surface of the adjacent tracheid, the microfibrils of which form rim (Figure 2h). The formation of boundaries during the S_2_ and S_3_ layers deposition makes the appearance of the pits in adjacent tracheids the same.

## 3. Discussion

### 3.1. The Proposed Formation Mechanism of a Pair of Bordered Pits in Tracheids

**Figure 3:**
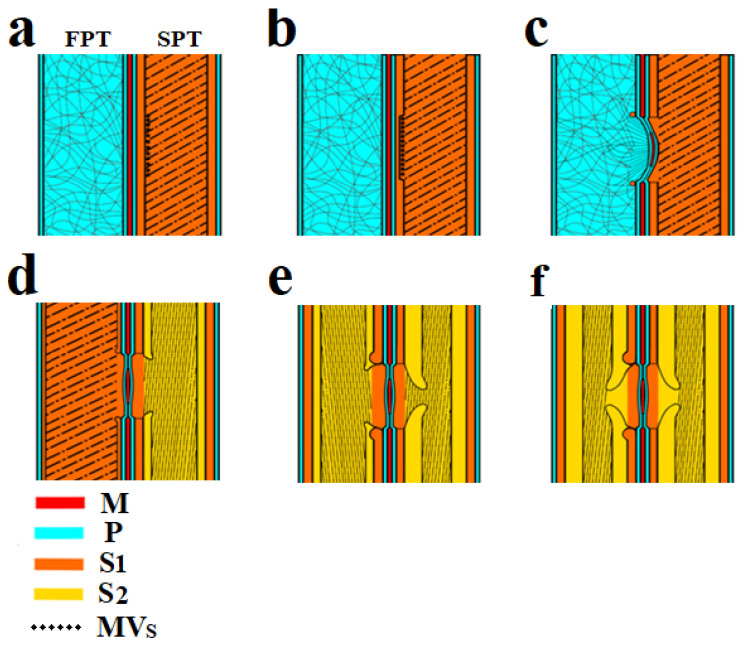
Scheme of bordered pit formation in the tracheid cell wall: (**a**) On the left (FPT) there is a tracheid with a primary wall, on the right (SPT)—a tracheid with a formed S_1_ layer of the secondary wall and exosomes localized on its surface. (**b**) Enzymatic destruction of the S_1_ layer under SPT endogluconase action occurs at the site of exosome localization. (**c**) The deformation of the P layers and torus occurs under the influence of greater pressure in the FPT. The formation of the S_1_ layer is observed as a rim around the pit. (**d**) The developing S_1_ layer in the FPT and S_2_ layer in the SPT. (**e**) Border deposition from the S_2_ layer in the SPT and FPT tracheids. (**f**) Final pit formation. *CM*—cellulose microfibrils, *S*_1_—S_1_ layer, *S*_2_—S_2_ layer, *P*—P layer, *M*—margo, *MVs—*multivesicular bodies, *SPT—*starting pit tracheid, *FPT*—following pit tracheid.

The development of the bordered pits in adjacent tracheids occurs asynchronously. The formation of a pit pair begins from the side of the initiator cell—starting pit tracheid (SPT) and continues with the adjacent cell—following pit tracheid (FPT). It should be noted that these tracheids are of different ages—the SPT is mature and already has a formed S_1_ layer, while the adjacent FPT tracheid is younger and only has the P layer. Based on the experimental data, a pit formation mechanism is presented (Figure 3), which includes several stages.

#### 3.1.1. Stage 1

The formation of bordered pits always occurs between two adjacent tracheids, and as a result of this process, a pit pair is formed. The initial stage is shown in Figure 2a,d and Figure 3a.

The SPT has a formed S_1_ layer of the secondary wall. A distinctive feature of the S_1_ layer is the specific microfibril slope angle of approximately 60° to the tracheid axis. This is confirmed by the SEM images in Figure 2b (55°), as well as previously obtained experimental data [13]. In these images, the S_1_ layer has already been formed, but pit formation has not started.

Carbohydrates, mainly starch, were completely consumed to form the S_1_ layer of the SPT. With the help of multivesicular bodies, the cell secretes vesicles in sites of future pit localization. The vesicles are spherical objects 100 nm in diameter (Figure 2d) containing enzymes. These extracellular vesicles are used to deliver endoglucanases to the surface of the S_1_ layer [13].

#### 3.1.2. Stage 2

Cellulose microfibrils are destroyed by the vesicle cellulase complex to form a cavity in the S_1_ layer (Figure 2f and Figure 3b).

Based on genome analysis, it was found that plant cellulase complex contains an incomplete cellulase pool. According to published data [30,31,32], the complex contains endo-1,4-β-glucanases (EG), cellobiases, and glucohydrolases but no cellobiohydrolases [31]. Endo-1,4-β-glucanases (EGs) are enzymes capable of cellulose destruction by breaking bonds in microfibrils. These enzymes can hydrolyse amorphous cellulose to gluco-oligosaccharides but cannot hydrolyse crystalline cellulose, xyloglucan, xylan, (1 → 3) (1 → 4) -β-D-glucan and other polysaccharides and oligosaccharides. The presence of EG has been shown in plant tissues in which cellulose formation takes place: differentiating xylem, phloem, cork, young and mature leaves, leaf veins, petioles, roots, and stem apices [32]. Secreted enzymes to degrade the cell wall have to be kept in place to limit their hydrolytic activity to the forming pit. This could be achieved either through interactions with integral plasma membrane proteins, or through direct attachment to the plasma membrane. Many secreted glucanases are anchored in the plasma membrane and may be localized through a specific lipid microenvironment [25].

Endoglucanase is unable to breakdown microfibrils of the primary wall, which are protected by pectin or xyloglucan and the enzymatic degradation stops. 

#### 3.1.3. Stage 3 

The next stage is the invagination of the two primary walls together with the intercellular substance (middle lamella) under the action of high pressure. Invagination occurs in the tracheid direction with the S_1_ layer (SPT). The invagination stage is shown in Figure 3c. Figure 2e shows a pit in which the effects of deformation caused by invagination are visible. As a result of this process, a randomly oriented network of microfibrils of the primary walls is stretched, and microfibrils are oriented from the pit periphery to the torus, forming a margo. The central part of the middle lamella remains unaffected and forms the pit torus.

Next, the synthesis of the S_1_ layer begins in the FPT. Cellulose microfibrils are synthesized by the cellulose-synthetase complex, which is a rosette structure located in the plasma membrane of a cell. The rosettes forming microfibrils are able to move freely along the membrane, but they are connected from the side of the cell wall with microfibrils, and on the other—with microtubules [33]. In fact, the observed microfibrils laid in the cell wall are rigid cylindrical rods with a diameter of about 30 nm. The orientation of the microfibrils changes so that they are concentrated around the formed obstacle (pit cavity). The rosette structures begin to deposit cellulose microfibrils around the circumference. As a result, a rim is formed around the pit (Figure 2e and Figure 3c) and then directly S_1_ layer (Figure 3d).

#### 3.1.4. Stage 4

The S_1_ layer formation is completed in the FPT (Figure 3d). The pressure in the adjacent tracheids is equalized, and the torus, together with cellulose microfibrils of the margo, occupies a central position, providing a free fluid flow with dissolved substances. This is necessary for delivery into the SPT of sucrose as a substrate for the formation of the S_2_ layer of the secondary wall.

The S_2_ layer is approximately eight times larger than the S_1_ layer of the secondary wall. The substrate for the formation of the S_2_ layer is delivered to the tracheids through the pits from the photosynthetic parts along the sieve elements of the phloem and then along the rays. The slope angle of S_2_ layer microfibrils is practically parallel to the tracheid axis. Cellulosic microfibrils have a certain rigidity and cannot pass over the formed cavity in the cell wall. Rosette structures deposit microfibrils bypassing the pits and forming a border overhanging the pit aperture. The diameter of the border is slightly less than the diameter of the torus. This is necessary for the correct functioning of the pit, that is, for the complete overlap of the aperture by the torus.

#### 3.1.5. Stage 5

The S_2_ layer in the FPT is formed according to the mechanism described in the previous stage for the SPT. It is possible that S_2_ layer formation in FPTs and SPTs can occur simultaneously. These two processes are categorized into separate stages in the scheme (Figure 3) to explain the new mechanism.

#### 3.1.6. Stage 6

At this stage, the processes associated with pit formation in the tracheids end.The formation of S_3_ and W (verrucous) layers of the secondary cell wall occurs (Figure 3f).

### 3.2. Analysis of the Proposed Mechanism in the Framework of the Microtubule Hypothesis

Proposed asynchronous mechanism for the formation of a bordered pits pair in tracheids is based on the stage of local enzymatic destruction of the S_1_ layer of one of the tracheids with the exosomes participation, invagination of two primary walls of adjacent tracheids together with the intercellular substance, and completion of the secondary cell wall formation in both tracheids. The intertracheid pit begins to form in a mature tracheid with an S1 layer due to local enzymatic hydrolysis by exosome-delivered endoglucanases in the proposed mechanism. The mechanism does not contradict the hypothesis of the microtubules role in the formation of the cellulose cytoskeleton, but takes into account the contribution of exosomes to the stage of pits pair formation. According to our mechanism, microtubules transport multivesicular bodies containing exosomes. The exosomes are released onto the inner surface of the cell wall where multivesicular bodies come into contact with the cell membrane. This determines the location of the pit.

According to the current microtubule hypothesis, heterogeneous deposition of cellulose occurs by a localized distribution of plasma membrane cellulose synthase complexes, in the movement of which cortical microtubules are involved. It is assumed that cellulose synthase complexes in the form of hexagonal rosettes containing CESA proteins and CESA-like proteins synthesize polysaccharides that comprise plant cell walls [33]. Microtubules can participate in this process, control the transport, and delivery of cellulose synthase complexes to the plasma membrane [23]. Funada et al. (1997) [34] investigated the relationship between bordered pit formation and microtubule localization, as Uehara and Hogetsu (1993) [22] proposed the involvement of microtubules in determining and maintaining the position of pit boundaries as one aspect of microtubule function in localized cell wall deposition. It has been suggested that the plexus of the MTs guides the vesicles that are very close to the microtubules [22,35,36]. 

The shape and localization of a pit pair, the mechanistic details regarding how microtubules and cellulose microfibrils work together to effect cell expansion remains controversial in the microtubule hypothesis [23]. 

Why during the formation of the primary wall, microtubules disappear locally in the circular areas in the regions where pits are formed, is not clear. How can circular bands of microtubules be involved in the deposition of concentrically oriented microfibrils at the pit boundaries? Uehara and Hogetsu (1993) [22] suggested that microtubules divide the cell surface into two domains, which activate and inactivate, respectively, the secondary wall deposition. However, it is unclear how this happens. 

In works [37,38] it is shown that during the formation of the S_1_ layer, microtubules are approximately oriented perpendicular to the long axis of the cell while microfibrils are strictly oriented parallel to each other. It is obvious that poorly ordered microtubules cannot control the stacking of strictly parallel microfibrils.

The published works do not show how the S_2_ layer is formed using microtubules. The synthesized microfibril, falling on the surface of the cell wall under construction, forms hydrogen bonds with the ligno-hemicellulose matrix and previously deposited microfibrils. We believe that in this case, the direction of packing of the synthesized microfibril is determined by the direction of the already deposited microfibrils. Microtubules in the liquid cytoplasm cannot influence the direction of packing of the microfibril fixed in the «solid» cell wall.

We believe that the influence of microtubules on the direction of microfibrils is possible only at the initial moment, when the microfibril is short and has not had time to form enough bonds with the cell wall surface. The change in the direction of microfibrils depositing is mainly associated with a change in the direction of previously deposited microfibrils. When a pit is formed, the microfibril appears in a free area and, in accordance with its rigidity, bends towards the nearest previously deposited microfibrils of the cell wall. The deposition can go in a circle, forming a pit border if the diameter of the pit is large. A slit-like pit is formed when the pit diameter is small. The microfibrils stiffness does not allow for greater bending. Microfibrils cover the pit cavity by slightly curving.

The process of pit formation in plants still has various questions unaddressed and unknown and opens up many interesting and new research opportunities.

## 4. Materials and Methods

### 4.1. Plant Materials

For this study, we harvested stem samples of a Norway spruce (*Picea abies* (L.) Karst.) growing on the border of its range in the Arkhangelsk region, 64.45° N, 40.94° E, Russia. The diameter of the spruce stem was 283 mm at 1.5 m above ground level. The use of mature trees (about 80 years old) with a large diameter is necessary in order to simultaneously observe several tracheids at different stages of development on radial sections. The study used the stem of one tree to work with samples of the same anatomical structure. Samples taken in different months (from April to October 2021) and at different stages of secondary vegetative growth were examined. We took two samples monthly. The most intense xylem formation was observed in the samples in August—therefore, it was these samples that were used to describe the stages of the pit formation in this work.

### 4.2. Sample Preparation, Treatment, and SEM Visualization

Wood cylinders (32 × 20 mm) from the stem of a living tree were removed using hole saws to obtain intact areas of developing xylem. After removal, the samples were immediately immersed in liquid nitrogen. The holes left in the tree after the removal of samples were covered with garden varnish. This technique made it possible to take several samples from one tree in different growing seasons without disrupting its functioning. The SEM sample preparation involving preliminary cryomechanical destruction and freeze drying was used [13,39]. This is a simple and quick pretreatment method that does not require chemical treatment and subsequent fixation of the samples in resin. The samples were fragmented using liquid nitrogen and immediately dried without thawing using a Labconco freeze dryer (FreeZone 2.5 L). To increase the image contrast of the samples, a gold–palladium coating with a thickness of 5 nm was applied to the surface of the wood sections using a Q150T ES (Quorum Technologies Ltd., Laughton, UK) sputter coater. Sample images were obtained using a Sigma VP Zeiss scanning electron microscope (Carl Zeiss Microscopy GmbH, Cambridge, UK) (10 kV accelerating voltage, InLens detector, 7.5 µm aperture).

### 4.3. Enzymatic Treatment

The sections of the formed (mature) xylem 10 × 7 × 2 mm in size, obtained by the cryomechanical method, were placed in Eppendorf tubes with the excessive enzyme concentration (more than 1000 units/mL) in acetate buffer pH 5 and incubated for 12 h at 20 °C. The enzyme preparations used were Pectinex Ultra SP-L from Novozymes (Kalundborg, Denmark), endoglucanase #1535.1.2 and xylanase #3-348.2H from the Research Center of Biotechnology RAS (Moscow, Russia). After enzyme treatment each sample was repeatedly washed with distilled water, frozen at −80 °C, and lyophilized. The surface of the dried sample was sputter-coated with a thickness of 5 nm, and SEM analysis was performed.

## 5. Conclusions

A new asynchronous mechanism for the formation of a bordered-pit pair in tracheids has been proposed. The mechanism explains the formation of the margo and torus of the bordered pit. The formation of bordered pits occurs between two adjacent tracheids, and as a result of this process, a pit pair is formed. The formation of a pit pair begins from the side of the initiator cell—the starting pit tracheid and continues with the adjacent cell—following pit tracheid. A new asynchronous mechanism for the formation of a pair of bordered pores in tracheids has been proposed.

The most important stages of pit formation are enzymatic destruction of the S_1_ layer at the site of the future pit on the one hand, invagination of two primary walls together with the intercellular substance on the other, and completion of secondary cell wall formation on both sides. The intertracheid pit begins to form in a tracheid with an S1 layer due to local enzymatic hydrolysis by exosome-delivered endoglucanases. The exosomes are released onto the inner surface of the cell wall where multivesicular bodies come into contact with the cell membrane. This determines the location of the pit.

## Data Availability

Not applicable.
